# Supplementary material for: Comparison and trends in outcomes of inborn and outborn infants born before 33 weeks’ gestation
Source: PLoS One. 2026 Apr 21;21(4):e0326648. doi: 10.1371/journal.pone.0326648 (PMC13098933; doi:10.1371/journal.pone.0326648)
Supplement: S2 Table — Adjusted odds ratios (OR) and its 95% confidence intervals (95% CI) were calculated after adjusting for confounders (gestational age, mode of delivery, presence or absence of intrauterine growth restriction, gender). aSevere IVH: grade ≥3 intraventricular hemorrhage. bPVL: periventricular leukomalatia, defined as presence of one or more cysts in the periventricular region of the brain, detected by serial ultrasound scans. cSevere ROP: stage ≥3 retinopathy of prematurity. dSurgical NEC: necrotizing enterocolitis requiring a surgical intervention (i.e., laparotomy, laparoscopy, intraperitoneal drain) due to intestinal perforation or failure to improve with medical management. eBPD: the need for supplemental oxygen at 36 weeks PMA or discharged on oxygen therapy whichever occurred first. fLOS: Late onset sepsis was defined as a culture of a pathogenic organism (bacterium or fungus) from blood and/or cerebrospinal fluid after 72 hours of age. gMorbidity-free survival: survival without any of these morbidities at term corrected age: BPD, surgical NEC, late-onset sepsis, PVL and severe IVH. See main text for detailed definitions of the individual morbidities. (DOCX) [file pone.0326648.s002.docx]

| **Outcome** | **adjusted OR (95% CI)**  **(ANS not included)** | **p-value** |
| --- | --- | --- |
| **Severe IVH^‡^** | 2.356 (1.293; 4.295) | 0.005 |
| **PVL^§^** | 1.578 (0.436; 5.716) | 0.487 |
| **Severe ROP^¶^** | 1.148 (0.405; 3.250) | 0.796 |
| **Surgical NEC^¥^** | 1.539 (0.503; 4.707) | 0.450 |
| **BPD^×^** | 0.918 (0.486; 1.733) | 0.792 |
| **LOS°** | 0.554 (0.263; 1.170) | 0.122 |
| **Mechanical ventilation** | 2.701 (1.812; 4.026) | <0.001 |
| **Morbidity free survival^$^** | 0.679 (0.439; 1.051) | 0.083 |
| **Mortality** | 1.496 (0.745; 3.006) | 0.257 |

**Supplementary Table 2: Analysing the influence of outborn status on neonatal outcomes using multivariable logistic regression model**

Adjusted odds ratios (OR) and its 95% confidence intervals (95% CI) were calculated after adjusting for confounders (gestational age, mode of delivery, presence or absence of intrauterine growth restriction, gender).

^a^Severe IVH: grade ≥3 intraventricular hemorrhage.

^b^PVL: periventricular leukomalatia, defined as presence of one or more cysts in the periventricular region of the brain, detected by serial ultrasound scans.

^c^Severe ROP: stage ≥3 retinopathy of prematurity.

^d^Surgical NEC: necrotizing enterocolitis requiring a surgical intervention (i.e., laparotomy, laparoscopy, intraperitoneal drain) due to intestinal perforation or failure to improve with medical management.

^e^BPD: the need for supplemental oxygen at 36 weeks PMA or discharged on oxygen therapy whichever occurred first.

^f^LOS: Late onset sepsis was defined as a culture of a pathogenic organism (bacterium or fungus) from blood and/or cerebrospinal fluid after 72 hours of age.

^g^Morbidity-free survival: survival without any of these morbidities at term corrected age: BPD, surgical NEC, late-onset sepsis, PVL and severe IVH. See main text for detailed definitions of the individual morbidities.
