# Supplementary material for: Comparison and trends in outcomes of inborn and outborn infants born before 33 weeks’ gestation
Source: PLoS One. 2026 Apr 21;21(4):e0326648. doi: 10.1371/journal.pone.0326648 (PMC13098933; doi:10.1371/journal.pone.0326648)
Supplement: S3 Table — Models included the dependent variable (outcome), birth location, antenatal steroid prophylaxis (ANS), and their interaction term (birth location × ANS). Odds ratios (OR) with 95% confidence intervals (CI) and p-values were calculated.a Severe IVH: intraventricular hemorrhage grade ≥3. b LOS: late-onset sepsis, defined as a culture of a pathogenic organism (bacterium or fungus) from blood and/or cerebrospinal fluid obtained after 72 hours of age. c Morbidity-free survival: survival at term corrected age without any of the following morbidities: BPD, surgical NEC, LOS, PVL, or severe IVH. See main text for definitions of individual morbidities. d ANS: antenatal steroid prophylaxis. (DOCX) [file pone.0326648.s003.docx]

**Supplementary Table 3. Focused logistic regression models including interaction term**

| **Outcome** | **Predictor** | **OR (95% CI)** | **p-value** |
| --- | --- | --- | --- |
| Severe IVH | birth location | 1.131 (0.491; 2.603) | 0.773 |
|  | ANS | 0.392 (0.204; 0.751) | 0.005 |
|  | interaction (birth location*ANS) | 0.716 (0.212; 2.421) | 0.591 |
| LOS | birth location | 2.156 (0.518; 8.984) | 0.291 |
|  | ANS | 1.240 (0.375; 4,104) | 0.725 |
|  | interaction (birth location*ANS) | 1.453 (0.245; 8.621) | 0.681 |
| Survival | birth location | 1.689 (0.756; 3.774) | 0.201 |
|  | ANS | 4.568 (2.545; 8.200) | <0.001 |
|  | interaction (birth location*ANS) | 0.919 (0.177; 4.77) | 0.920 |
| Morbidity free survival | birth location | 1.230 (0.645; 2.346) | 0.530 |
|  | ANS | 1.645 (1.027; 2.634) | 0.038 |
|  | interaction (birth location*ANS) | 1.611 (0.681; 3.81) | 0.277 |

Models included the dependent variable (outcome), birth location, antenatal steroid prophylaxis (ANS), and their interaction term (birth location × ANS). Odds ratios (OR) with 95% confidence intervals (CI) and p-values were calculated.

a Severe IVH: intraventricular hemorrhage grade ≥3.
b LOS: late-onset sepsis, defined as a culture of a pathogenic organism (bacterium or fungus) from blood and/or cerebrospinal fluid obtained after 72 hours of age.
c Morbidity-free survival: survival at term corrected age without any of the following morbidities: BPD, surgical NEC, LOS, PVL, or severe IVH. See main text for definitions of individual morbidities.
d ANS: antenatal steroid prophylaxis.
